# A framework for heart-lung interaction and its application to prone position in the acute respiratory distress syndrome

**DOI:** 10.3389/fphys.2023.1230654

**Published:** 2023-08-07

**Authors:** Jon-Emile S. Kenny

**Affiliations:** ^1^ Health Sciences North Research Institute, Sudbury, ON, Canada; ^2^ Flosonics Medical, Toronto, ON, Canada

**Keywords:** heart-lung interactions, hemodynamics, prone position, acute respiratory distress syndrome, venous return, fluid responsiveness

## Abstract

While both cardiac output (Q_circulatory_) and right atrial pressure (P_RA_) are important measures in the intensive care unit (ICU), they are outputs of the system and not determinants. That is to say, in a model of the circulation wherein venous return and cardiac function find equilibrium at an ‘operating point’ (OP, defined by the P_RA_ on the x-axis and Q_circulatory_ on the y-axis) *both* the P_RA_ and Q_circulatory_ are, necessarily, *dependent* variables. A simplified geometrical approximation of Guyton’s model is put forth to illustrate that the *independent* variables of the system are: 1) the mean systemic filling pressure (P_MSF_), 2) the pressure within the pericardium (P_PC_), 3) cardiac function and 4) the resistance to venous return. Classifying independent and dependent variables is clinically-important for therapeutic control of the circulation. Recent investigations in patients with acute respiratory distress syndrome (ARDS) have illuminated how P_MSF_, cardiac function and the resistance to venous return change when placing a patient in prone. Moreover, the location of the OP at baseline and the intimate physiological link between the heart and the lungs also mediate how the P_RA_ and Q_circulatory_ respond to prone position. Whereas turning a patient from supine to prone is the focus of this discussion, the principles described within the framework apply equally-well to other more common ICU interventions including, but not limited to, ventilator management, initiating vasoactive medications and providing intravenous fluids.

## Introduction

Though evidence of benefit has existed for placing patients with moderate-to-severe acute respiratory distress syndrome (ARDS) in the prone position for some time, the coronavirus pandemic raised clinical awareness of this maneuver (Guérin et al., 2020). Guidelines currently recommend prone position for patients with ARDS and a partial pressure-to-fraction of inspired oxygen (P_a_O_2_/F_i_O_2_) ratio of not more than 150 mmHg (Papazian et al., 2019). Furthermore, with this ARDS severity, patients should maintain the prone position for at least 12 h per day for optimal benefit (Guérin et al., 2013).

Turning a patient from the supine to prone position has salutary benefits on gas exchange as oxygenation and carbon dioxide elimination are both enhanced (Guérin et al., 2020). The mechanisms by which the prone position exerts its salubrious effects are manifold. When the dorsal, de-gassed ‘sponge lung’ (Bone, 1993) is no longer gravity-dependent, it is recruited and the surface area for gas exchange increased. Critically, the newly-enlisted alveoli see no significant change in pulmonary blood flow (Henderson et al., 2013); as a consequence, the burden of low ventilation-to-perfusion (V/Q) lung units is reduced. In addition to alveolar recruitment, shifting to the prone position improves ‘shape matching’ between the pulmonary parenchyma and the chest wall (Gattinoni et al., 2013). In total, the result is that there is less pulmonary inhomogeneity (Cressoni et al., 2014; Cressoni et al., 2015) and, therefore, fewer ‘stress-raisers’ (Mead et al., 1970) that amplify radial traction forces upon the lungs *and* pulmonary vasculature (Broccard et al., 1998; Marini et al., 2003; Repessé et al., 2016). Furthermore, stiffening the chest wall with improved pulmonary compliance diminishes trans-pulmonary pressure (P_TP_) as the pleural pressure is raised for any given airway pressure (Marini and Gattinoni, 2021). This reduces the mechanical power applied to the pulmonary parenchyma and mitigates West zone 1 and 2 conditions (Gattinoni and Quintel, 2016). All of the aforementioned changes in pulmonary physiology (i.e., improved oxygenation and carbon dioxide elimination, optimized perivascular pulmonary mechanics, diminished P_TP_) minimize the afterload experienced by the right ventricle (RV), giving weight to the motto: ‘what’s good for the lung is good for the RV (Repessé et al., 2016).’

While the literature is replete with elegant investigations into the mechanical pulmonary pathophysiology of ARDS in both supine and prone positions, comparatively little is known about the hemodynamic effects. With a recent investigation exploring the determinants of venous return in the prone position (Lai et al., 2021) and an excellent related review (Lai et al., 2023), this overview will expand upon relevant concepts in clinical hemodynamics, propose a simplified geometrical model clarifying the determinants of cardiac output and right atrial pressure and then relate this to what is currently known about prone position in the ARDS patient (Table 1).

**TABLE 1:** Key messages by section.

Introduction	A cursory overview of the mechanical effects of prone position (PP) on the injured lung. PP recruits both airspace and pulmonary vasculature. This improves pulmonary mechanics, gas exchange and reduces right ventricular outflow impedance
Guyton primer	The *venous return* (VR) subsection describes the: 1.) pressure gradient for VR (i.e., P_MSF_—P_RA_), 2.) resistance to VR (R_VR_) and 3.) how both (P_MSF_—P_RA_) and R_VR_ together describe VR.
	The *Guyton diagram* subsection describes how VR and cardiac function form an equilibrium—the operating point (OP)—which is *the dependent variable* in the Guyton model. As the OP is a dependent variable, so too are its two coordinates (i.e., P_RA_ and Q_circulatory_). Thus, contrary to what is commonly taught, P_RA_ is not an independent determinant of Q_circulatory_ (i.e., total circulatory blood flow = venous return = cardiac output)
Geometrical model	To illustrate how P_RA_ is not a determinant of Q_circulatory_, a simplified geometrical model is derived; the independent variables of the circulation are shown to be: 1.) P_MSF,_ 2.) the pericardial pressure (P_PC_), 3.) R_VR_ and 4.) ‘cardiac resistance’ (R_cardiac_)
	The circulation can be ‘cardiac-’ or ‘venous-limited.’ The former is synonymous with preload unresponsiveness. When the circulation is ‘cardiac-limited’, changing P_MSF_ or R_VR_ only alters P_RA_ with no effect on Q_circulatory_. When ‘venous-limited’, changing cardiac function (R_cardiac_) or P_PC_ only alters P_RA_ with no effect on Q_circulatory_
Implications for prone position	Recent investigations in ARDS patients report how PP alters P_MSF_, R_VR_, and R_cardiac_; little data exist on how PP alters the circulation via the P_PC_ (which is a key nexus for heart-lung interaction)
	Determining a ‘cardiac-limited’ circulation helps predict the hemodynamic response to PP.
	In response to PP, P_RA_ does not determine Q_circulatory_, the system (as described by the geometrical model) determines the OP which decides both Q_circulatory_ *and* P_RA_.

## Guyton primer

Many excellent reviews connecting Guyton’s model of the circulatory system to critical-illness are available (Sylvester et al., 1983; Bressack and Raffin, 1987; Fessler, 1997; Jacobsohn et al., 1997; Magder, 2004; Gelman, 2008; Parkin and Leaning, 2008; Feihl and Broccard, 2009a; Feihl and Broccard, 2009b; Magder, 2012; Berlin and Bakker, 2015; Berger and Takala, 2018; Persichini et al., 2022). Though this model has been criticized and debated (Brengelmann, 2003; Beard and Feigl, 2011; Moller et al., 2017; Berger et al., 2019; Brengelmann, 2019; Werner-Moller et al., 2020; Kenny, 2021), these controversies are beyond the scope of this review. Guyton’s contributions to hemodynamics are many and may be parsed into: 1.) the explication of venous return (Guyton et al., 1955) and 2.) the graphical superposition of the venous return and Starling-Sarnoff curves (Guyton, 1955).

### Venous return

The determinants of venous return from the peripheral circulation are, from Guyton’s experiments: 1.) the mean circulatory filling pressure (P_MCF_), 2.) right atrial pressure (P_RA_) and 3.) the resistance to venous return (R_VR_) (Guyton, 1955; Sylvester et al., 1983; Jacobsohn et al., 1997; Feihl and Broccard, 2009a). Together the P_MCF_ and the P_RA_ define the pressure gradient for venous return.

#### The pressure gradient for venous return

If blood flow were ceased, arterial pressure would fall and venous pressure would rise to a weighted recoil pressure reflecting the portion of the circulation with greatest blood volume (Sylvester et al., 1983; Jacobsohn et al., 1997). As the small veins and venules comprise this circulatory segment, the P_MCF_ is a ‘pivot pressure’ found downstream of the capillary beds but upstream from the larger veins (Magder, 2012). The ‘pivot pressure’ description arises from the sense that when the heart recommences circulatory flow, pressure in the arteries rise up from the P_MCF_ while the pressure in the downstream veins fall below it; thus, the P_MCF_ acts as a quasi-static ‘pivot’ around which pressures upstream and downstream rise and fall, respectively (Broccard, 2012). As discussed below, the P_MCF_ is similar, but not equivalent to, the mean *systemic* filling pressure (P_MSF_). The P_MSF_ excludes the contributions of intrathoracic blood volume and compliance.

**FIGURE 1:**
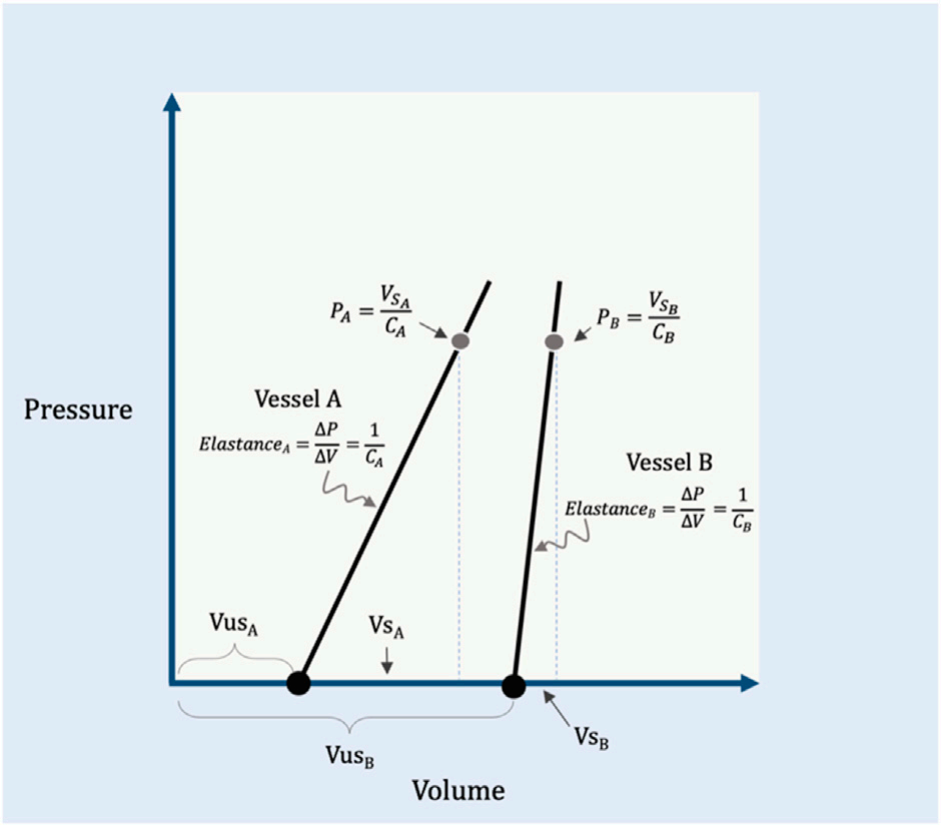
Illustration of capacitance and compliance. Vessel A has a relatively small capacitance because its unstressed volume (V_US_A__) is small. The compliance of the vessel (C_A_) is the inverse of elastance on this graph; by rearrangement, the recoil pressure generated in this vessel (P_A_) is equal to its stressed volume (V_S_A__) divided by its compliance. Vessel B shows a larger capacitance, but an increased elastance (i.e., reduced compliance, C_B_) relative to vessel A. Vessel A and B are analogous to the ‘water balloon’ and ‘waterbed,’ respectively, described within the text. V_S_B__ is the stressed volume, V_US_B__ is the unstressed volume and P_B_ is the recoil pressure of vessel B.

The P_MCF_ (or P_MSF_) is determined by two related–and often confused–biophysical properties: capacitance and compliance (Rothe, 1986; Rothe, 1993; Tyberg, 2002). To understand *capacitance*, the reader must appreciate that the total circulatory volume is comprised of two distinct (though dynamic) ‘types’ of volume–the unstressed (V_US_) and stressed (V_S_) volumes (Gelman, 2008; Magder, 2012). The V_US_ does not create a vascular elastic recoil pressure while the V_S_ does. As an analogy, filling a waterbed requires water volume before the walls are stretched (i.e., the V_US_); further volume generates a recoil pressure from the elastic walls (i.e., the V_S_). As compared to a water balloon, a waterbed has a much larger capacitance because its V_US_ is greater than the V_US_ of the balloon. *Compliance* and its inverse, *elastance*, describe the relationship between changing vascular volume and changing recoil pressure (Rothe, 1993). It follows that compliance (or elastance) pertain to the V_S_; the V_US_, by definition, generates no change in pressure (i.e., the V_US_ has infinite compliance or zero elastance). Continuing with the analogy above, were the waterbed made from a poorly elastic (i.e., stiff) material, it would have a large capacitance, but low compliance (or high elastance). If the water balloon was made of a highly elastic material, it would have a low capacitance, but high compliance (or low elastance). Mathematically, the P_MCF_ is determined by the volume of blood generating a recoil pressure (i.e., the V_S_, which is determined by total vascular volume and capacitance) divided by the vascular compliance (Tyberg, 2002; Magder, 2012) (Figure 1).

**FIGURE 2:**
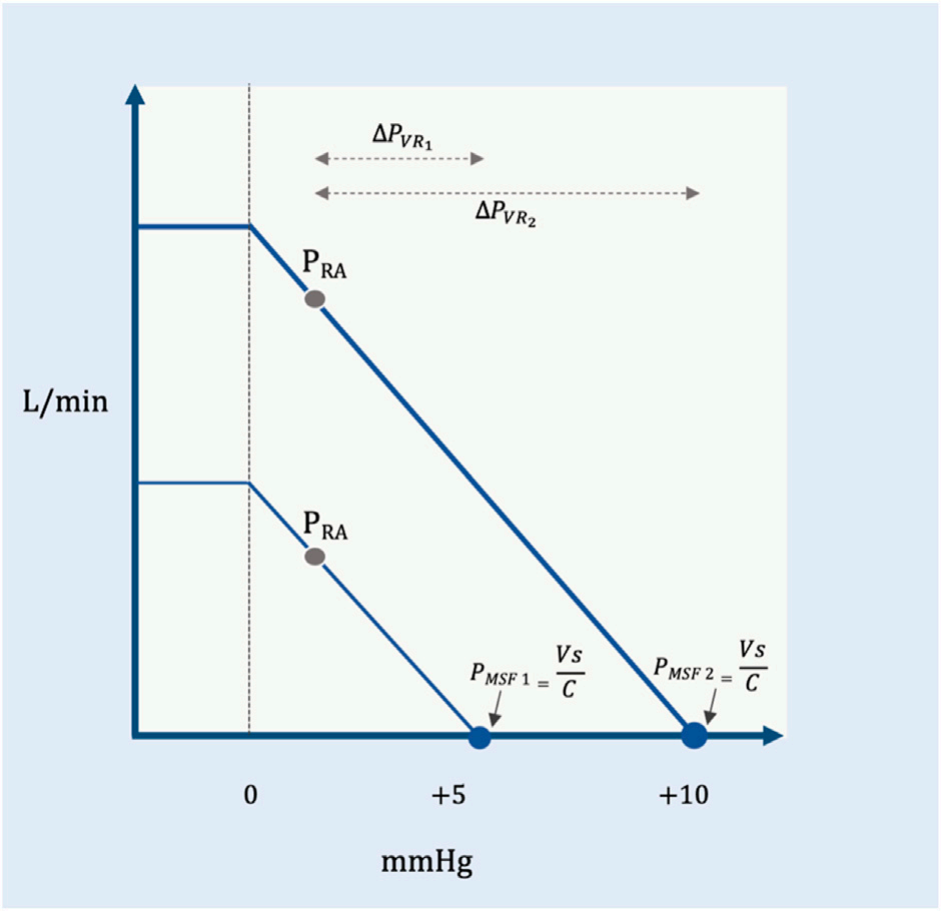
Pressure gradient for venous return. The effect of changing mean systemic filling pressure (in millimeters of mercury, mmHg) from a low (P_MSF1_) to a higher value (P_MSF2_) (e.g., volume infusion, decreased capacitance). The slope of the venous return curve is constant between the two curves meaning that the resistance to venous return is constant (see below). For a given right atrial pressure (P_RA_), the lower P_MSF1_ (i.e., reduced pressure gradient for venous return, ∆ P_VR1_) causes a diminished venous return on the y-axis (liters per minute, L/min). The same P_RA_ in a system with P_MSF2_ (i.e., increased pressure gradient for venous return, ∆ P_VR2_) results in a higher venous return on the y-axis. The P_MSF_ is the pressure in the right atrium at zero flow (i.e., the x-intercept). V_S_ and C are the stressed volume and average compliance, respectively, of the systemic vasculature. The flattening of the venous return curve is where the great veins collapse; this creates a maximal venous return in each state.

As noted above, the P_MCF_ includes the cardiac and pulmonary vascular volumes and compliances (i.e., the total circulation) while the P_MSF_ measures only the extra-thoracic, systemic, circulation (Rothe, 1993); they are very similar in value and often used interchangeably. In clinical practice, the methods for estimating this static, ‘pivot pressure’ reflect the *systemic* pressure (i.e., P_MSF_) and this measure will be used throughout this review (Berger et al., 2016). For patients, there are three methods to estimate the P_MSF_: 1.) extrapolation to zero flow of the P_RA_–cardiac output relationship altered by ventilator-hold maneuvers (Pinsky, 1984; Maas et al., 2009), 2.) extremely rapid cuff insufflation on the arm with an ipsilateral arterial line (Maas et al., 2012) and 3.) mathematical modelling by the method of Parkin and Leaning (Parkin and Leaning, 2008). Though beyond the scope of this discussion, the ventilator-hold and arm-occlusion methods over-estimate P_MSF_ (Maas et al., 2012; Berger et al., 2016) for a variety of reasons (Moller and Berger, 2023) whereas the mean systemic filling pressure analogue (P_MSA_) (i.e., the method of Parkin and Leaning) accurately estimated absolute and changing values of P_MSF_ in a porcine model (Werner-Moller et al., 2022). Because it is simply calculated from P_RA_, cardiac output and mean arterial pressure (Parkin and Leaning, 2008; Moller and Parkin, 2022), the P_MSA_ is an attractive tool for guiding both prospective and retrospective research as well as clinical therapy (Moller and Parkin, 2022; Moller and Berger, 2023). Given the above, the importance of understanding and, arguably, measuring the P_MSF_ is that it is a hemodynamic variable the clinician can target therapeutically. For example, a low P_MSF_ intimates low V_S_ which could be due to diminished total blood volume (e.g., hypovolemia, hemorrhage) and/or high venous capacitance (e.g., venodilation, sepsis). The clinician might rectify these pathological states by giving volume and/or administering alpha-agonists, respectively (Parkin and Leaning, 2008). Thus, the P_MSF_ and its determinants are independent variables that can be adjusted for therapeutic control of the circulation; increasing P_MSF_ raises venous return for any given right atrial pressure (P_RA_) (Figure 2).

Downstream from the P_MSF_ is the P_RA_. In Guyton’s original experimental set-up, P_RA_ was studied as an independent variable, altered via the height of a collapsible tube (Guyton et al., 1957). Guyton observed that the P_RA_ was inversely related to venous return; in other words, decreasing P_RA_ increased venous return, linearly (Figure 2). Consequently, the difference between P_MSF_ and P_RA_ is the pressure gradient for venous return ( ∆ P_VR_); the value of this gradient is directly proportional to blood return to the right heart (Equation 1). More concretely, an increase in P_MSF_ and/or decrease in P_RA_ will augment venous return and *vice versa* (Magder, 2012).

#### The resistance to venous return

**FIGURE 3:**
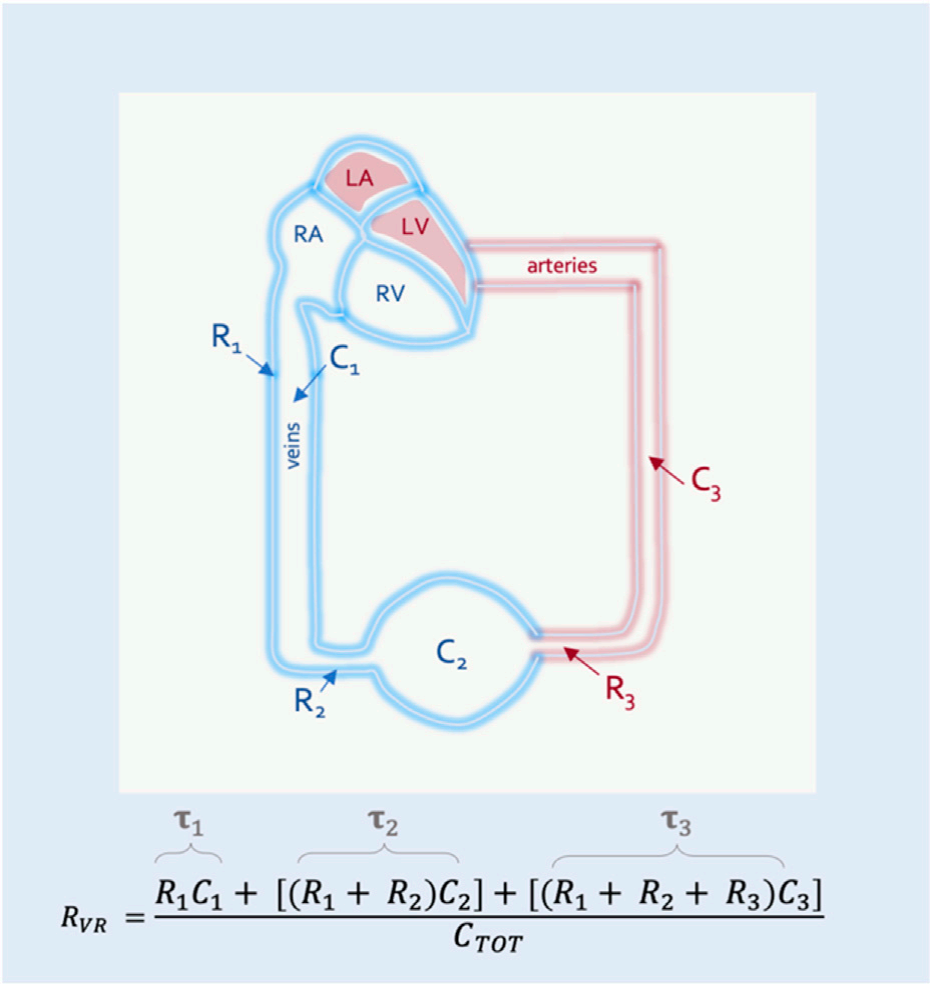
The resistance to venous return (R_VR_). R_VR_ is composed of resistances (R_x_) and compliances (C_x_) for the entire circulation. This simplified model shows 3 vascular segments in series. The R_VR_ is the sum of the resistance and compliance for each segment divided by the total compliance of the circulatory system (C_TOT_). Resistance multiplied by compliance is the time constant ( τ ).

Guyton began with a mathematical approximation of the circulation, modeled after a system of distensible tubes (Jacobsohn et al., 1997). In this representation, the forces that resist total blood flow back to the heart are termed the ‘resistance to venous return’ (R_VR_). While the R_VR_ is often considered to be a purely Poiseuillean description of the venous circulation, this is not correct. The R_VR_, like the P_MSF_, is a weighted average of the system (i.e., including arterial components) (Jacobsohn et al., 1997). Each vascular bed faces a downstream resistance and has a unique compliance; the R_VR_ is a summation of the downstream resistance encountered by each vascular bed, multiplied by its individual compliance relative to the total compliance of the system (Figure 3).

**FIGURE 4:**
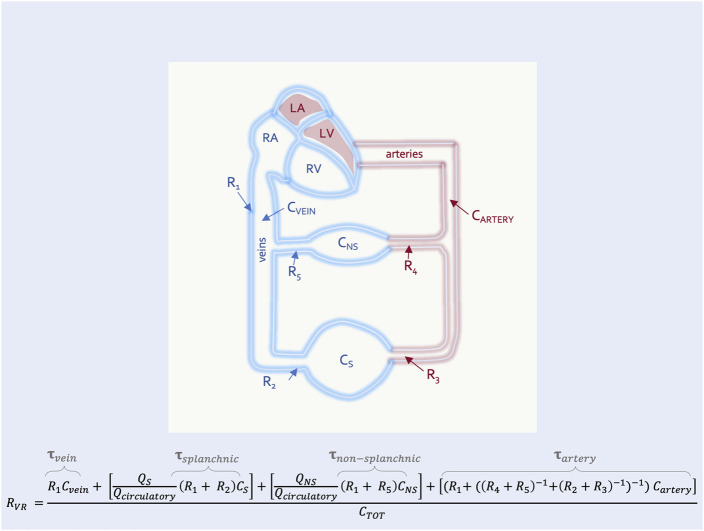
The resistance to venous return with high and low time-constant segments in parallel. This is an expansion of Figure 3 with two representative segments in parallel–the non-splanchnic (NS) (i.e., low time constant, $\left.\tau \right)$ and splanchnic (S) (i.e., high $\left.\tau \right)$ segments. Here the fraction of total circulatory flow (Q_circulatory_) to the splanchnic (i.e., Q_S_/Q_circulatory_) *versus* non-splanchnic (i.e., Q_NS_/Q_circulatory_) segments determines the R_VR_. If all blood diverted to the splanchnic segment (i.e., Q_S_/Q_circulatory_ = 1.0; Q_NS_/Q_circulatory_ = 0.0), its higher compliance (C_S_) increases R_VR_. If all blood diverted to the non-splanchnic segment (i.e., Q_S_/Q_circulatory_ = 0.0; Q_NS_/Q_circulatory_ = 1.0), its lower compliance (C_NS_) decreases R_VR_ (assuming all other resistances remain equal). C_TOT_ is the total compliance of the system.

In this way the R_VR_ can also be described by the time constant (i.e., the resistance multiplied by the compliance) of each vascular segment (Magder, 2016). This is clinically-important because diverting blood volume towards or away from a vascular bed with a long time constant (e.g., the splanchnic circulation) will increase or decrease the R_VR_, respectively (Caldini et al., 1974). The converse is true for vascular beds with a short time constant (e.g., kidneys, muscle) (Magder, 2016) (Figure 4). Accordingly, should an intervention in the ICU (e.g., prone position) alter the fraction of flow to vascular beds of differing time constants, R_VR_ will be affected.

**FIGURE 5:**
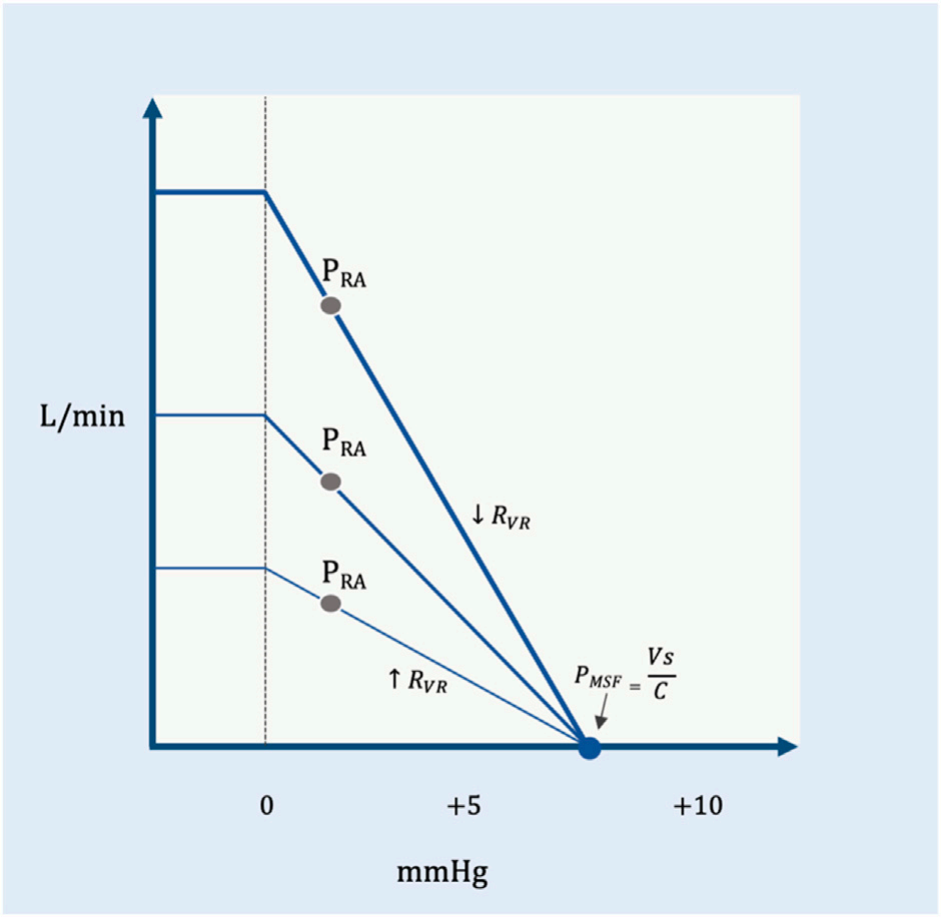
The R_VR_ and the venous return curve. P_MSF_ is constant, but the resistance to venous return changes. The shallow curve is a higher resistance ( ↑ R_VR_) the steeper curve is a lower resistance ( ↓ R_VR_). At the same P_RA_, lower resistance and higher resistance generate increased and decreased flow (L/min), respectively. V_S_ and C are the stressed volume and average compliance, respectively, of the systemic vasculature. The flattening of the venous return curve is where the great veins collapse; this creates a maximal venous return in each state.

On the venous return curve, change in the R_VR_ alters the slope for a given pressure gradient (Figure 5). An increase in R_VR_ reduces the slope, while a decrease in R_VR_ steepens the slope.

In summary, venous return is directly proportional to the P_MSF_ less the P_RA_ and indirectly proportional to the R_VR_. If the P_MSF_ increases and/or P_RA_ falls, then venous return rises (Figure 2). Similarly, decreased R_VR_ facilitates blood return to the heart and *vice versa* (Figure 5). The Ohmic representation of this relationship is as follows (Berger and Takala, 2018):

$$venous\;return=\frac{P_{MSF}-P_{RA}}{R_{VR}}$$ (1)

### Venous return and cardiac function: the Guyton diagram

**FIGURE 6:**
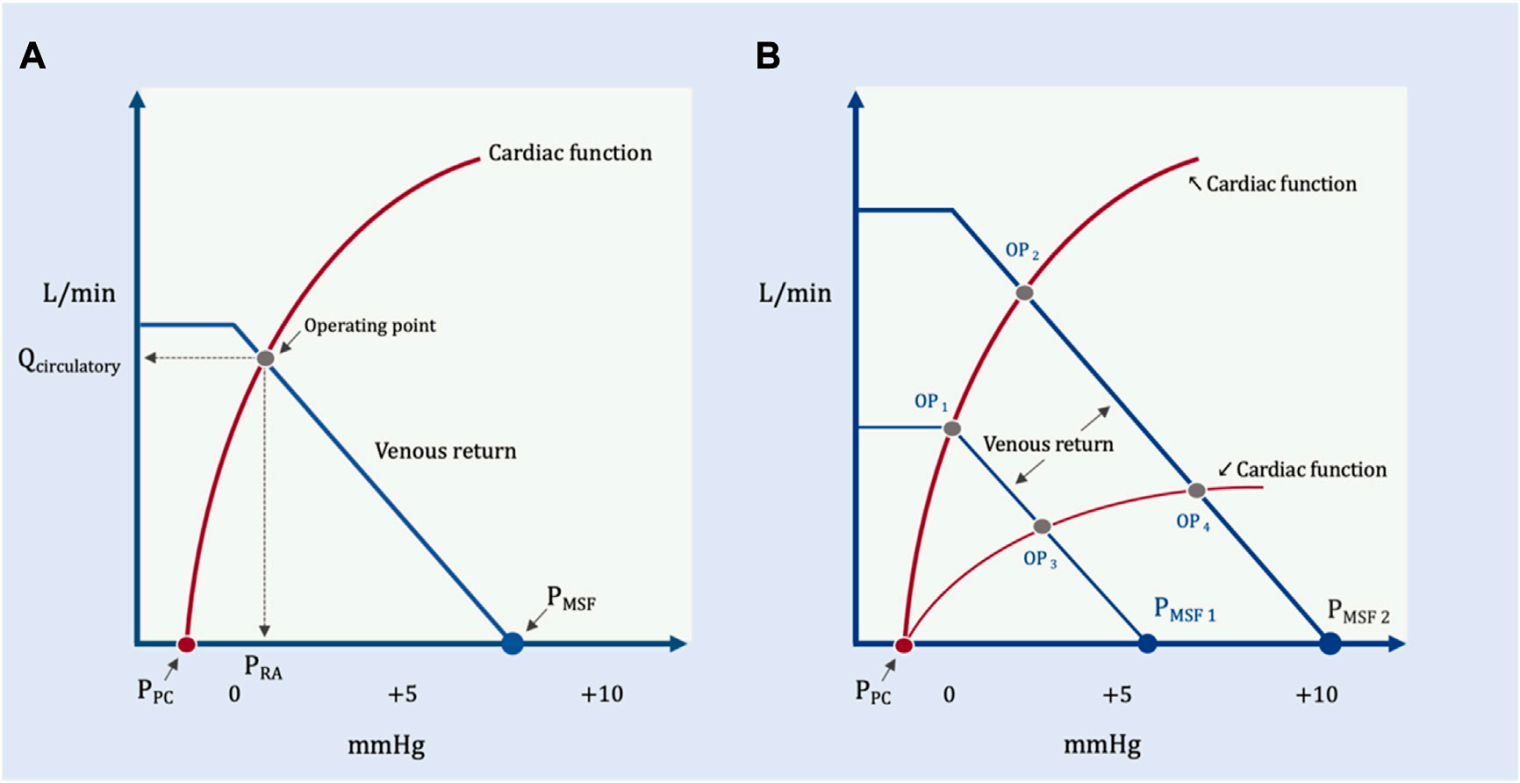
The Guyton diagram. **(A)** The circulation in its resting state; as in Figure 2, the x-axis is right atrial pressure in millimeters of mercury (mmHg) and y-axis is total blood flow in liters per minute (L/min). The P_MSF_ is approximately 8 mmHg at the x-intercept of the venous return curve (in blue). The Starling-Sarnoff (or cardiac function) curve is in red in a normal, upright position; its x-intercept is the pressure around the right atrium, the pericardial pressure (P_PC_). In this model, the dependent variable is the operating point, at the intersection of the venous return and cardiac function curves at equilibrium. Accordingly, both the x- (i.e., P_RA_) and y- (i.e., Q_circulatory_) coordinates defined by the operating point are also dependent variables. **(B)** How the P_RA_ and Q_circulatory_ are determined by the system. Normal cardiac function but diminished P_MSF1_ (e.g., volume loss, venodilation) results in operating point 1 (OP_1_), diminished P_RA_ and Q_circulatory_. Normal cardiac function with increased P_MSF2_ (e.g., volume expansion, decreased venous capacitance from adrenergic agents) causes OP_2_ (i.e., increased P_RA_ and Q_circulatory_). Reduced P_MSF_ and diminished cardiac function (e.g., acute cor pulmonale with tricuspid regurgitation) leads to OP_3_. Elevated P_MSF_ with reduced cardiac function leads to OP_4_. Both Q_circulatory_ and P_RA_ are dependent variables in this system. The independent variables are reflected in the position and slopes of the venous return and cardiac function curves.

In addition to detailing the peripheral vascular determinants of blood returning *to* the heart, Guyton expanded our understanding of hemodynamics by adding to his analysis the cardiac determinants of blood flow *from* the heart. He did so by superimposing the venous return and Starling-Sarnoff curves (Guyton, 1955); this depiction is commonly referred to as the ‘Guyton diagram.’ These curves can be placed over each other because they both have P_RA_ on the x-axis and blood flow on the y-axis (Figure 6). Though it will be developed in more detail below, the Guyton diagram introduces an important distinction between intravascular and transmural pressures. The P_RA_ and P_MSF_ measured on the Guyton diagram are intravascular pressures. Thus, the pressure gradient for venous return is directly related to the difference between the *intravascular* P_MSF_ and P_RA_ (Equation 1). The Starling mechanism, however, is related to right atrial *transmural* pressure which is the pressure within the right atrium less its ambient pressure (i.e., the pericardial pressure). The transmural right atrial pressure is a static pressure that determines cardiac myocyte stretch which servo-controls the ejected stroke volume to match the venous return inflow.

Like any model, the value of the Guyton diagram is that it makes explicit the system’s independent and dependent variables. Independent variables are those things the clinician can change or control (e.g., vascular volume and capacitance, airway pressure), whereas dependent variables are what the clinician wants to predict or study (e.g., cardiac output) by manipulating the independent variables. These distinctions are critical when considering the effects of any intervention in the ICU (e.g., prone position).

Nestled within the venous return curve are some of the independent variables of the circulatory system touched upon above: 1.) vascular capacitance, 2.) total vascular volume and 3.) the R_VR_. Thus, increasing total vascular volume via intravenous fluids and/or decreasing vascular capacitance via alpha-agonists both augment the V_S_ and, therefore, P_MSF_. On the Guyton diagram, raising P_MSF_ right-shifts the venous return curve such that there is increased blood flow to the heart for any given P_RA_. Similarly, beta-agonists (Green, 1977) and/or shunting blood from long to short time-constant vascular beds decreases the R_VR_ (Caldini et al., 1974); this also enhances venous return for any given P_RA_. On the Guyton diagram, diminished R_VR_ is manifested by an increased slope of the venous return curve (Figure 6). The converse also holds, diminished blood volume, increased capacitance and/or increased R_VR_ all reduce venous return for any given P_RA_. One clinically-important scenario wherein vascular capacitance rises (i.e., which decreases P_MSF_) is reduced adrenergic tone (e.g., sedation, anesthesia, relief of hypoxemia) (Bressack and Raffin, 1987).

Found within the cardiac function curve are additional independent variables: heart rate, rhythm, valve function, afterload, inotropic and lusitropic states (Feihl and Broccard, 2009a; Feihl and Broccard, 2009b). Consequently, rate and rhythm control (e.g., cardioversion), afterload reduction (e.g., vasodilator therapy, pulmonary vascular recruitment), enhanced contractility and improved relaxation (e.g., epinephrine infusion) all increase the slope of the Starling-Sarnoff curve. With this, blood flow from the heart is enhanced for any given P_RA_. The converse also holds, for example, rapid atrial dysrhythmia coupled with torrential tricuspid regurgitation and severe pulmonary arterial hypertension decreases the slope of the cardiac function curve, that is to say, reduce cardiac output for any given P_RA_ (Figure 6).

But what about the P_RA_ itself? Is it an independent variable? In Guyton’s experimental work on venous return, P_RA_ was studied as an independent variable. However, on the Guyton diagram, which considers both venous return and cardiac function simultaneously, P_RA_ is *not* independent. This was clearly stated by Guyton in his initial proposal: “*right atrial pressure is not one of the primary determinants of cardiac output but, instead, is itself determined simultaneously with cardiac output*” (Guyton, 1955). Later, Fiehl and Broccard expanded upon P_RA_ as a dependent variable in their excellent review (Feihl and Broccard, 2009a). Accordingly, when analyzing venous return and cardiac function *simultaneously*, the dependent variable is the equilibrium formed at their intersection–the operating point. Thus, both the x- (i.e., P_RA_) and y- (i.e., cardiac output) Cartesian coordinates are equally dependent upon the system. This may be counterintuitive given the convention of placing the independent variable on the x-axis, however, with the Guyton diagram this is a vestige of his initial work on venous return. When it is understood that the operating point is the dependent variable, the circular and specious reasoning that the concept of venous return is incorrect because ‘raising P_RA_ reduces venous return per Guyton but augments cardiac output by Starling’ becomes moot. Rather, at any given time (or in response to an intervention, such as the prone position) there are characteristics of the peripheral circulation and heart that, in tandem, produce a unique cardiac output *and* P_RA_ (Guyton, 1955). To clarify this, a modified Guyton model is proposed below to disclose the clinically-relevant independent variables.

## A geometrical model

**FIGURE 7:**
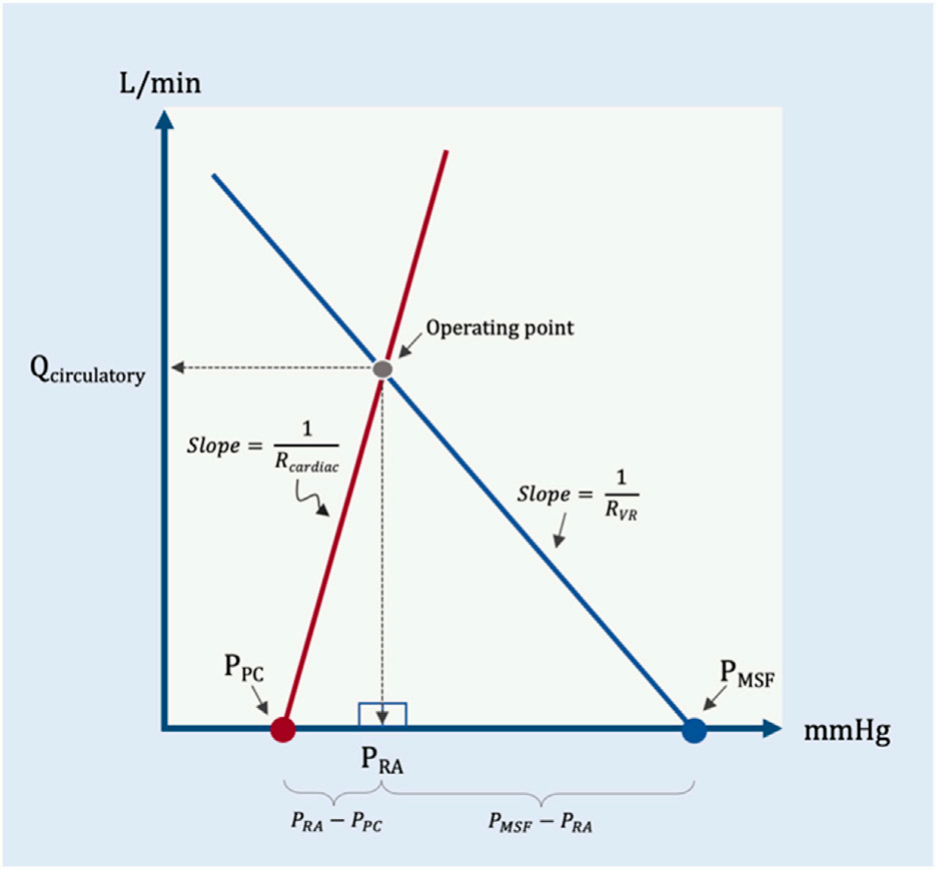
Simplified geometrical model. This model borrows from the Guyton diagram where the red line represents cardiac function and the blue line venous return. Two right triangles are formed as described in the text; the operating point is the apex of the two right triangles. Note that the slope (change in flow per unit pressure) is conductance, G. The inverse of conductance is resistance. As in previous figures, P_PC_ is pericardial pressure, P_MSF_ is mean systemic filling pressure, R_cardiac_ and R_VR_ are cardiac and venous resistance, respectively. Q_circulatory_ is blood flow of the system with right atrial pressure (P_RA_) in millimeters of mercury (mmHg) on the x-axis and blood flow in liters per minute (L/min) on the y-axis.

This is a simplified geometric approximation of the principles discussed above. If we consider the intersection of cardiac function and venous return as two directly-opposed right triangles, then we can solve for the height of their shared apex at equilibrium (i.e., cardiac output or venous return presently identified as Q_circulatory_) as a function of their bases and hypotenuse slopes (Figure 7). Q_circulatory_ is numerically equivalent to cardiac output and/or venous return. It is used in the geometrical model to emphasize that total blood flow (i.e., Q_circulatory_) is determined by the operating point–the intersection of both peripheral venous *and* cardiac function. This avoids the confusion that sometimes arises when ‘cardiac output’ is thought to be determined only by cardiac factors or when ‘venous return’ is thought entirely due to peripheral factors; ‘Q_circulatory_’ circumvents this ambiguity.

The base of the left triangle rests on the x-axis and is defined by the pressure immediately surrounding the heart, within the pericardium (i.e., the P_PC_) and the P_RA_; this is the transmural pressure of the right atrium. The slope (i.e., hypotenuse) of this triangle is the change in cardiac output per mmHg of transmural right atrial pressure, or cardiac conductance (G_cardiac_). This value is estimated to be 35 mL/min/kg per 1 mmHg (Rothe, 1993). Multiplying the base of this triangle (i.e., P_RA_–P_PC_) by the slope of the hypotenuse (G_cardiac_) gives the height of this triangle (i.e., total circulatory flow, Q_circulatory_).

$$Q_{circulatory}=G_{cardiac}\;x\;\left(P_{RA}-P_{PC}\right)$$ (2)

Equation 2 is solved for P_RA_

$$P_{RA}=\frac{Q_{circulatory}}{G_{cardiac}}+P_{PC}$$ (3)

Similarly, the base of the rightmost triangle is defined by the P_MSF_ and the P_RA_; this is the pressure gradient for venous return (the difference between two intravascular pressures along a hypothetical length of vessel), as above. The slope of this triangle is the change in cardiac output per the gradient for venous return, or venous conductance (G_VR_). Based on a P_MSF_ of 8 mmHg, this value is estimated to be 10 mL/kg/min per 1 mmHg. Multiplying the base of this triangle (i.e., P_MSF_–P_RA_) by the slope of its hypotenuse (G_VR_) gives the height of this triangle, which is also total circulatory flow, Q_circulatory_.

$$Q_{circulatory}=G_{VR}\;x\;\left(P_{MSF}-P_{RA}\right)$$ (4)

Equation 4 is solved for P_RA_

$$P_{RA}=P_{MSF}-\frac{Q_{circulatory}}{G_{VR}}$$ (5)

Setting equation 3 equal to equation 5, we can reduce the equation to Q_circulatory_ as follows:

$$P_{MSF}-\frac{Q_{circulatory}}{G_{VR}}=\frac{Q_{circulatory}}{G_{cardiac}}+P_{PC}$$ (6)

$$\frac{P_{MSF}}{Q_{circulatory}}-\frac{1}{G_{VR}}=\frac{Q_{circulatory}}{G_{cardiac}}+P_{PC}$$ (7)

$$\frac{P_{MSF}-P_{PC}}{Q_{circulatory}}=\frac{1}{G_{cardiac}}+\frac{1}{G_{VR}}$$ (8)

$$Q_{circulatory}=\frac{P_{MSF}-P_{PC}}{\frac{1}{G_{cardiac}}+\frac{1}{G_{VR}}}$$ (9)

Because the inverse of conductance, G, is resistance, this equation can be written as:

$$Q_{circulatory}=\frac{P_{MSF}-P_{PC}}{R_{cardiac}+R_{VR}}$$ (10)

**FIGURE 8:**
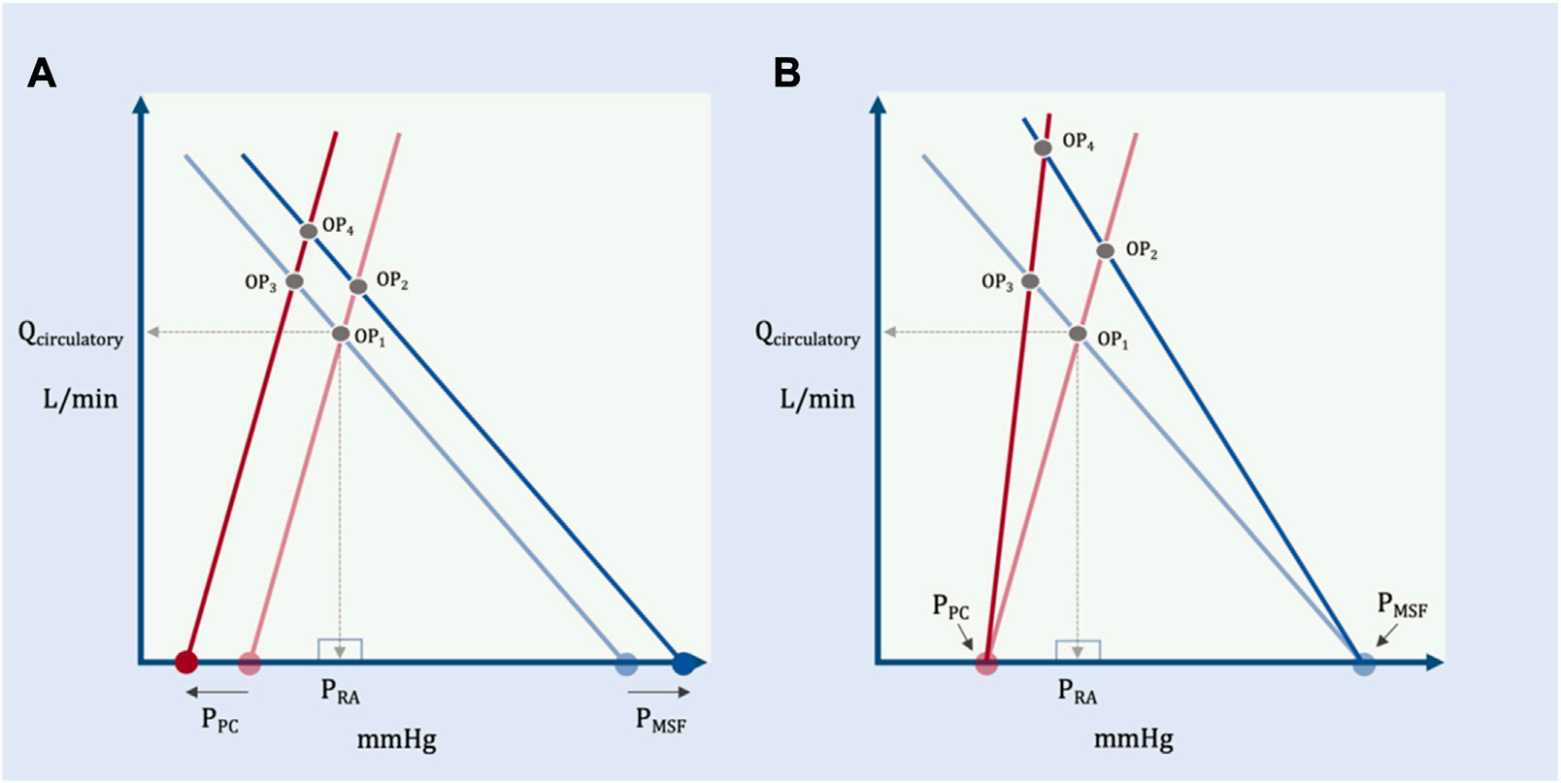
The independent and dependent variables of the geometric model. **(A)** The effect of changing the independent variables, P_PC_ and P_MSF_ on the dependent variable (operating point, OP). OP_1_ depicts baseline conditions, its x- (P_RA_) and y-(Q_circulatory_) coordinates are shown. A solitary increase in P_MSF_ (e.g., volume infusion) results in OP_2_, that is, increased P_RA_ and Q_circulatory_. A selective decrease in P_PC_ (e.g., spontaneous inspiration) leads to OP_3_ which increases Q_circulatory_, but decreases P_RA_. If P_MSF_ rises and P_PC_ falls, the result is OP_4_, increased Q_circulatory_ at a slightly reduced P_RA_ relative to baseline. **(B)** The effect of changing the independent variables, R_VR_ and R_cardiac_ on the dependent variable (operating point, OP). A selective decrease in venous resistance (e.g., shunting blood away from the splanchnic circulation) leads to OP_2_ (i.e., both P_RA_ and Q_circulatory_ rise). A selective decrease in cardiac resistance (i.e., improving cardiac function by, for example, reducing pulmonary vascular resistance) leads to OP_3_ (i.e., P_RA_ falls, while Q_circulatory_ rises). Reducing venous and cardiac resistance together leads to OP_4_ (i.e., little P_RA_ change with large Q_circulatory_ augmentation).

Accordingly, in this model the shared apex of the two triangles (i.e., the operating point, which defines Q_circulatory_) is a function of the total base of the two triangles (i.e., P_MSF_ less P_PC_) and the inverse of the slopes of their respective hypotenuses (i.e., R_VR_ and R_cardiac_). More concretely, if R_VR_ and R_cardiac_ remain constant, increased P_MSF_ and/or decreased pressure surrounding the heart (P_PC_) raise the height of their shared apex (Figure 8). A concomitant decrease in R_cardiac_ (i.e., increased slope of the Starling-Sarnoff curve) or R_VR_ (i.e., increased slope of the venous return curve) would further elevate their shared apex (Figure 8).

In this model, P_RA_ plays no role in cardiac output because the operating point (i.e., the shared apex) is the dependent variable; Q_circulatory_ and P_RA_ *both* fall out from this equilibrium (Feihl and Broccard, 2009a). The equations above could have equally been solved for P_RA_ instead of Q_circulatory_; P_RA_, nevertheless, would still be dependent upon P_MSF_, P_PC_, R_cardiac_ and R_VR_.

To further develop this model with an emphasis on heart-lung interaction, the determinants of P_PC_ are included. Doing so reveals additional, clinically-relevant independent variables when placing an ARDS patient in the prone position. The P_PC_ is the x-intercept of the hypotenuse defined by R_cardiac_ (i.e., the cardiac function curve) (Magder, 2004; Feihl and Broccard, 2009a). As originally hypothesized by Guyton (Feihl and Broccard, 2009a) and demonstrated by Marini and colleagues (Marini et al., 1981), increasing P_PC_ initiates a parallel, right-shift of the cardiac function curve. Consequently, increased P_PC_ decreases the shared apex (i.e., the operating point) and Q_circulatory_ is diminished but only if there is no simultaneous change in P_MSF_, R_cardiac_ or R_VR_.

Given that the P_PC_ is a summation of: 1.) pleural pressure (P_PL_), 2.) pressure added by mechanical ventilation (i.e., estimated as the mean airway pressure, P_AW_, multiplied by the ratio of the chest wall to respiratory system elastances, E_CW_/E_RS_) (Gattinoni et al., 2004) and 3.) the elastic recoil pressure of the pericardium (P_PC_EL__) (Cabrera et al., 1989), we can expand equation 10 above.

$$Q_{circulatory}=\frac{\left.P_{MSF}-{\left[P\right.}_{PL}+\left(P_{AW}∙\frac{E_{CW}}{E_{RS}}\right)+P_{{PC}_{EL}}\right]}{R_{cardiac}+R_{VR}}$$ (11)

Accordingly, increased pleural (e.g., thoracic supports) and/or elastic recoil pressure from the pericardium (e.g., right ventricular dilatation in acute cor pulmonale), raise the pressure surrounding the heart, P_PC_. Furthermore, elevated P_AW_ (e.g., increasing positive end-expiratory pressure, PEEP) or a stiffened chest wall (e.g., prone position increases the E_CW_/E_RS_ ratio) both amplify P_PC_; from equation 11, we see that increasing P_PC_ reduces Q_circulatory_ but only if P_MSF_, R_cardiac_ and R_VR_ are constant. It should not escape the reader’s attention that including P_PC_ in this model is a crucial link between cardiac and respiratory physiologies.

### Cardiac limitation

**FIGURE 9:**
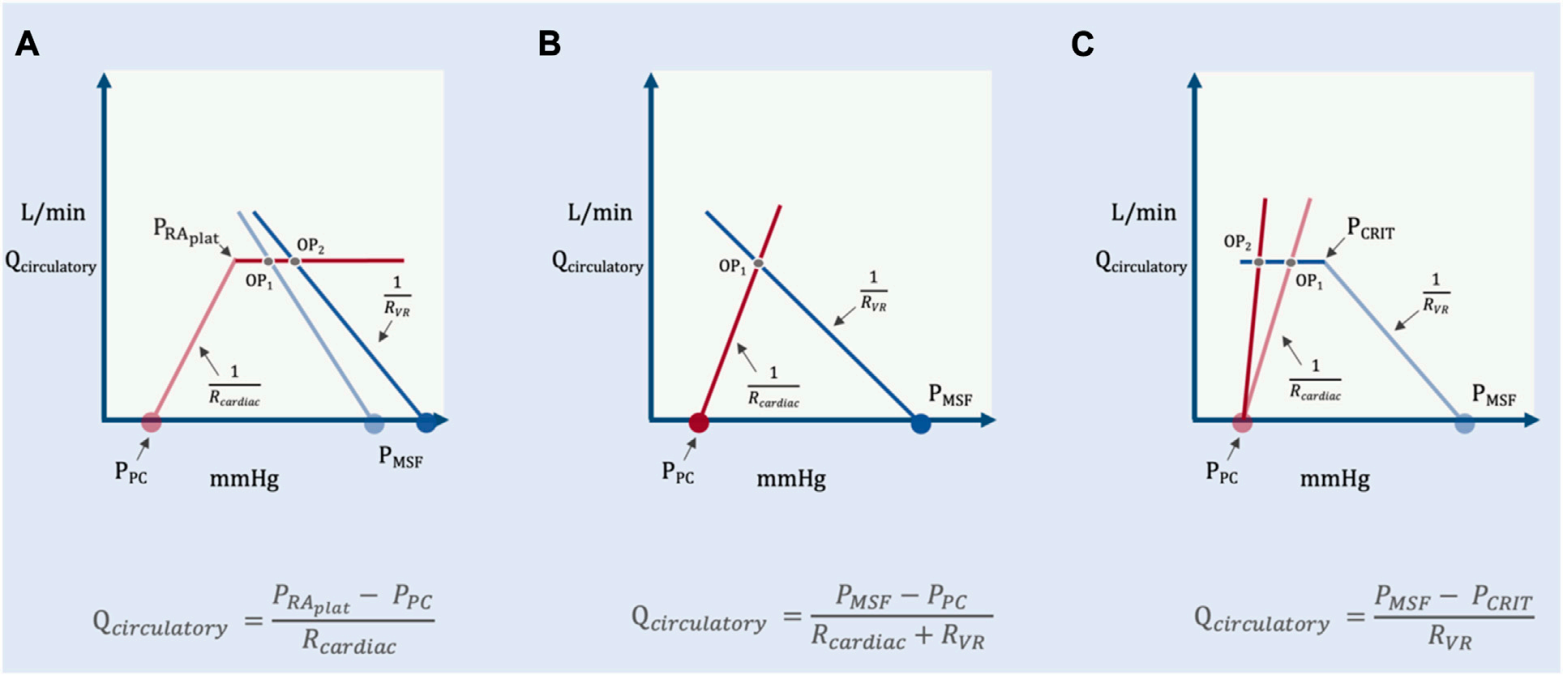
Operating point positions in the geometrical model. **(A)** reveals cardiac limitation where the operating point (OP) is on the flat portion of the cardiac function curve. Change in P_MSF_ or R_VR_ change only P_RA_ and not Q_circulatory_ (OP_1_ *versus* OP_2_). **(B)** is when the system is neither venous nor cardiac limited. Q_circulatory_ is changed by P_MSF_, P_PC_, R_cardiac_ and R_VR_ (see Figure 5). **(C)** shows venous limitation or ‘waterfall’ physiology. Changes in cardiac function alter only P_RA_ and not Q_circulatory_ (OP_1_ *versus* OP_2_).

While the proposed model is meant to illuminate the clinically-relevant independent variables determining Q_circulatory_, equation 11 has important caveats (Magder, 2012). The most important is that it is predicated upon the intersection of two hypotenuses; *in vivo*, both the venous return and cardiac function curves have portions that flatten out. When the operating point falls upon the flat portion of the cardiac function curve, Q_circulatory_ depends only upon the independent variables of cardiac function: P_PC_, the right atrial pressure at which the cardiac function curve begins to plateau, P_RAplat_ and the R_cardiac_ (Figure 9).

$$Q_{circulatory}=\frac{P_{{RA}_{plat}}-P_{PC}}{R_{cardiac}}$$ (12)

Fundamentally, this equation relays that Q_circulatory_ is no longer determined by peripheral factors when the operating point is above the P_RAplat_. Changing P_MSF_ or R_VR_ only alter P_RA_ with fixed Q_circulatory_.

### Venous limitation

In a manner similar to cardiac function, the venous return curve also flattens when the P_RA_ falls below venous collapse pressure, P_CRIT_ (Magder, 2012). This is the formation of a Starling resistor when the great veins enter the thorax and is observed with ultrasound as collapse of the great veins. This phenomenon is also termed ‘waterfall’ physiology because the pressure below P_CRIT_ has no bearing on flow, just as the height of a waterfall does not mediate its flow (Permutt and Riley, 1963). Consequently, when the operating point lies to the left of P_CRIT_ (i.e., on the “flat portion” of the venous return curve) Q_circulatory_ becomes independent of cardiac function or P_PC_; the independent variables are P_CRIT_, P_MSF_ and R_VR_ (Figure 9).

$$Q_{circulatory}=\frac{P_{MSF}-P_{CRIT}}{R_{VR}}$$ (13)

In other words, when venous limited, reducing R_cardiac_ (i.e., improving cardiac function) or changing P_PC_ has no bearing on Q_circulatory_; only changing P_MSF_, R_VR_ or P_CRIT_ might alter total flow.

## Implications for the prone position

With a Guyton-based circulatory model proposed above, anticipating the change in Q_circulatory_ follows the independent variables of the system: P_MSF_, P_PC_, R_cardiac_ and R_VR_. At present there are three key studies that have elucidated interactions between the circulation and prone position in ARDS (Vieillard-Baron et al., 2007; Jozwiak et al., 2013; Lai et al., 2021). Much of the discussion below is taken from these investigations.

### Mean systemic filling pressure

Recently, Lai and colleagues studied the effect of prone position on the determinants of venous return (Lai et al., 2021). They measured P_MSF_ by extrapolating to zero flow a series of P_RA_–cardiac output pairings in response to increasing airway pressure. Though this method overestimated P_MSF_ in a porcine model (Berger et al., 2016), this observation was restricted to euvolemic conditions which are less likely in ARDS patients in the ICU. Nevertheless, considering the discussion on P_MSF_ measurement above, a retrospective calculation of P_MSA_ would be of great interest given that the average P_MSF_ measured by Lai et al. was clinically quite high, especially in the prone position. Irrespective of absolute values, Lai and colleagues observed that P_MSF_ increased significantly from the semi-recumbent to prone position; they hypothesized that this was due to increased intra-abdominal pressure (IAP). However, the baseline value and change in IAP had no bearing on P_MSF_ behavior. This is unsurprising given what is known about the mechanisms by which PEEP increase P_MSF_. Initially, it was also hypothesized that IAP mediated P_MSF_ augmentation with PEEP application and/or stiffening of the chest wall (i.e., akin to prone position) in early canine models (Scharf et al., 1977). However, IAP had no role in raising P_MSF_, instead, adrenergic reflexes (i.e., changing vascular capacitance) and redistribution of blood volume from the central to peripheral circulation were the main drivers of P_MSF_ rise (Scharf and Ingram, 1977; Fessler, 1995; Fessler, 1997). Accordingly, central blood volume, adrenergic reserve and exogenous vasoactive agents all undoubtedly mediate the change in P_MSF_ upon pronation, rather than IAP. Parenthetically, this could also explain hemodynamic differences noted between elective surgical and critically-ill ARDS patients when prone position is employed (Edgcombe et al., 2008). The latter are more likely to be on vasoactive agents and volume-loaded, while the former more likely euvolemic; as well, anesthetic agents may blunt reflexive changes in vascular capacitance which would limit P_MSF_ rise in the operating room. As described above, P_MSF_ is directly related to Q_circulatory_ when the patient is not cardiac limited and without concurrent changes in P_PC_, R_cardiac_ or R_VR_.

### Pericardial pressure

There are no known direct measurements of P_PC_ in humans with ARDS placed in the prone position. Yet, inferences can be made given the mathematical approximation of P_PC_ presented above. The prone position increases the elastance (i.e., stiffness) of the chest wall (E_CW_) (Pelosi et al., 1998). To the extent that pronation also decreases the elastance (i.e., improves compliance) of the lungs by alveolar recruitment, the E_CW_ relative to the elastance of the respiratory system (i.e., the lungs and the chest wall together, E_RS_) rises. Multiplying the mean airway pressure generated by mechanical ventilation by the E_CW_/E_RS_ ratio approximates P_PC_ augmentation when a patient is passive with the ventilator. For example, if the mean airway pressure is 10 mmHg with an E_CW_/E_RS_ ratio of 0.3 in the supine position, then 3 mmHg is added to the P_PC_. If mean airway pressure remains constant and prone position increases the E_CW_/E_RS_ ratio to 0.5, then 5 mmHg is added to the P_PC_.

Additionally, pericardial restraint could play an important role determining P_PC_, especially if there is comorbid acute cor pulmonale (ACP). Typically, when right atrial volume is low (i.e., estimated by a transmural pressure below 5 mmHg (Hamilton et al., 1994)), there is little recoil pressure generated by the pericardium around it. As atrial volume increases beyond this, the pericardial sac is engaged and moves up its volume-pressure relationship. This leads to an increasingly large elastic recoil pressure from the pericardium, which raises the P_PC_. Elevated P_PC_, therefore, restricts right ventricular filling and ‘protects’ from overdistention; however, this blunts Q_circulatory_ by narrowing the P_MSF_–P_PC_ gradient.

In the setting of ACP, often seen in moderate-to-severe ARDS (Guérin and Matthay, 2016; Mekontso Dessap et al., 2016), pericardial recoil may play an important role upon prone position. With ACP, co-existent right atrial distension elevates P_PC_ by pericardial recoil; this is especially true with P_RA_ above 10–12 mmHg (Hamilton et al., 1994). While prone position is expected to further increase P_PC_ (i.e., by increasing P_PL_), to the extent that the elevated P_PL_ shrinks cardiac volume, P_PC_ may remain constant, or even fall, as the elastic recoil pressure imparted by the pericardium is reduced. More simply, the rising P_PL_ experienced by the pericardial space is offset by falling recoil pressure of the pericardium. This was originally observed in models of continuous positive airway pressure in heart failure (Huberfeld et al., 1995). Were this to occur upon prone position in a patient with ACP, P_PC_ would remain constant or fall. Taken with the effect of prone position on P_MSF_ noted above, the P_MSF_–P_PC_ gradient would be maintained (or enhanced) and so too would Q_circulatory_ if R_cardiac_ and R_VR_ remain constant.

Finally, some have argued for the execution of prone position with thoracoabdominal supports that allow the abdomen to hang freely (Chiumello et al., 2006). These supports are typically placed mid-sternum and below the pelvis. Chiumello and colleagues compared these supports to the abdomen flush with the bed in prone ARDS patients (Chiumello et al., 2006). They found that the supports accentuated local pressure without any benefit to gas exchange while diminishing stroke volume. Given support placement directly at the sternum, it is possible that P_PC_ is accentuated, reducing the P_MSF_–P_PC_ gradient and Q_circulatory_ barring a concomitant decrease in R_cardiac_ or R_VR_.

### Cardiac resistance

While not a commonly-employed term within the sphere of clinical hemodynamics, ‘cardiac resistance’ (R_cardiac_) is analogous to R_VR_. Graphically and mathematically, R_cardiac_ is simply the inverse slope of the cardiac function curve. A decrease in R_cardiac_ (i.e., a steeper slope of the cardiac function curve) represents improved cardiac function and raises the operating point (i.e., Q_circulatory_) unless the system is venous limited. In an elegant ultrasonographic study, Vieillard-Baron and colleagues illuminated the salubrious effects on the RV prompted by prone position (Vieillard-Baron et al., 2007). In 21 patients with P_a_O_2_/F_i_O_2_ ratio of less than 100 mmHg and ACP defined as RV enlargement and septal dyskinesia, 18 h of prone position led to a significant reduction in heart rate and increase in cardiac output. Furthermore, RV end-diastolic area fell while LV end-diastolic area increased and tricuspid regurgitation was reduced. Taken together, the rise in cardiac output with diminished RV size strongly implies reduced R_cardiac_ as a mechanism of improved Q_circulatory_, at least in patients with ACP. The mechanism for this improvement (detailed at the outset of this review and by others (Repessé et al., 2016)) was reduced pulmonary vascular impedance to flow facilitating RV ejection (Jardin and Vieillard-Baron, 2003; Vieillard-Baron et al., 2007) which improves stroke volume and cardiac output for any given P_RA_.

Recent studies also imply reduced R_cardiac_. Ruste and colleagues investigated the hemodynamic effects of prone position in over 100 patients (Ruste et al., 2018). 25% of prone sessions led to significantly increased cardiac output, while 23% had a significant decrease; the remainder showed no change. Importantly, of those sessions where cardiac output rose, 56% had no change or a decrease in global end-diastolic volume (GEDV) measured by transpulmonary thermodilution. Rising cardiac output without an increase in end-diastolic volume infers reduced R_cardiac_. Importantly, static GEDV with prone position could signify a shrinking RV end diastolic volume with enlarging LV end diastolic volume consistent with the reduced RV-to-LV end-diastolic area ratio observed with echocardiography by Vieillard-Baron et al. (Vieillard-Baron et al., 2007). Finally, Boesing and colleagues recently published on different PEEP titration strategies and their interaction with prone position (Boesing et al., 2022). In this study, esophageal pressure (P_ES_) was used as a surrogate for P_PL_. Curiously, the PEEP titration strategy that led to the greatest increase in cardiac output from supine to prone was associated with the smallest rise in transmural P_RA_ (i.e., P_RA_ less P_ES_), in other words, the least preload augmentation. Similar to the observations by Ruste and colleagues, this finding suggests, but does not prove, enhanced cardiac function (i.e., reduced R_cardiac_).

### Resistance to venous return

In the study of Lai and colleagues (Lai et al., 2021), the R_VR_ was calculated from semi-recumbent to prone position in ARDS patients. In total, R_VR_ increased in the vast majority, though there were a few with stable or slightly diminished R_VR_. Like P_MSF_, the change in R_VR_ was not related to IAP and like P_MSF_, this is unsurprising given the foundational work of Takata and Robotham (Takata et al., 1990). In their original model, Takata and Robotham proposed that the relationship between great vein pressure and IAP would behave analogously to West zones in the lung. That is, if the IAP is much greater than inferior vena cava (IVC) pressure (i.e., zone 2), then venous return is impaired when the abdomen is pressurized by diaphragmatic descent and, in theory, prone position. However, if IAP is much less than IVC pressure (i.e., zone 3), then increased IAP generated by diaphragmatic descent (or prone position) enhances venous return. Their initial work confirmed this model, however, they later found that the model held even with an open abdomen and evisceration, that is, constant IAP (Takata and Robotham, 1992). Thus, the ambient pressure of import was more likely focal subcostal, crural, or intra-hepatic pressure, rather than general IAP. This was observed by Decramer and colleagues (Decramer et al., 1984) and explored further by Brienza et al. in a porcine model (Brienza et al., 1995) and Jellinek et al. in humans (Jellinek et al., 2000). Accordingly, diaphragmatic shape-matching between the liver and upper abdomen, active *versus* passive diaphragm displacement, intra-hepatic compliance (e.g., intrinsic liver disease) and the use of focal thoracoabdominal supports, among other factors might affect hepatic pressure (P_hepatic_) upon pronation. Diminished venous pressure (e.g., hypovolemia, venodilation) relative to P_hepatic_ might increase R_VR_. By contrast, elevated venous pressure (e.g., high blood volume, low venous capacitance) relative to P_hepatic_ might blunt a rise in R_VR_ with prone positioning.

Another possible mechanism for increased R_VR_ with prone position follows that of P_MSF_. As described above, reflex sympathetic tone is a key mediator of increased P_MSF_. However, when alpha agonists act upon veins to increase the V_S_, resistance necessarily rises. This is because change in volume is proportional to the second power of vessel diameter but resistance is related to the fourth power. More concretely, if the diameter of a vein falls by 20% from its baseline, its volume is diminished by 36% (i.e., this reduces its capacitance, increases P_MSF_) but its resistance rises by 244% (Rothe, 1993). Because the splanchnic circulation is a crucial reservoir for venous blood, the rise in resistance in response to V_S_ recruitment can be offset by beta-agonism (Green, 1977) in the hepatic veins, or redistribution of blood flow to short time constant vascular beds, as noted above (Magder, 2016) (Figure 4). Nevertheless, hepatosplanchnic blood flow during prone position in ARDS changes little (Hering et al., 2002; Matejovic et al., 2002). Interestingly, one study found decreased renal blood flow (Hering et al., 2001)—a fast time-constant bed; diversion of blood in this manner contributes to increased R_VR_. A final, potential mechanism for R_VR_ augmentation with prone position lies in the superior vena cava (SVC). Fessler found that the rise in total R_VR_ following PEEP application was predominantly due to the veins draining into the SVC rather than the IVC (Fessler et al., 1992). Because P_PL_ is the pressure that surrounds SVC and prone tends to raise P_PL_ for any given P_AW_ (see equation 11 above), it is possible that mechanical compression of the SVC contributes to R_VR_ (Lansdorp et al., 2014; Berger et al., 2016). Regardless of the mechanism, R_VR_ is a critical determinant of Q_circulatory_ (Pinsky, 2021).

### Knowing the limits

Taking the above into consideration, a key factor when predicting the hemodynamic response to prone position is the location of the operating point whilst semi-recumbent; is the operating point ‘cardiac limited’, ‘venous limited’ or ‘unlimited’ (Figure 6) (Magder, 2012)? Knowing this focuses the clinician on the independent variables most likely affecting Q_circulatory_. For instance, if the operating point is cardiac limited (Figure 6) we see that changes in P_MSF_ and R_VR_ play no role, while changes in cardiac characteristics (e.g., R_cardiac_) mediate Q_circulatory_. Of course, this depends on how close the operating point is to the P_RA_ at which the cardiac function curve flattens out, but this is, nevertheless, a reasonable clinical heuristic. Jozwiak and colleagues studied 18 ARDS patients with elevated right ventricular-to-left ventricular end-diastolic areas (RVEDA/LVEDA), but without ACP (Jozwiak et al., 2013). Prior to prone position, the change in cardiac output in response to a passive leg raise was evaluated. By the model above, ‘cardiac limitation’ is detected when a patient is preload unresponsive. In this state, only improved cardiac function during pronation (i.e., reduced R_cardiac_) increases Q_circulatory_; changes in P_MSF_ and R_VR_ shift the operating point along the x-axis, but not the y-axis. In other words, P_RA_ changes but not blood flow. Jozwiak and colleagues found that in ‘cardiac limited’ patients, prone position significantly reduced pulmonary vascular resistance and the RVEDA/LVEDA which should diminish R_cardiac_ and improve Q_circulatory_. However, these patients were also found to have depressed left ventricular ejection fraction. Furthermore, in the face of prone position, systemic afterload increased; total R_cardiac_, therefore, did not improve.

**FIGURE 10:**
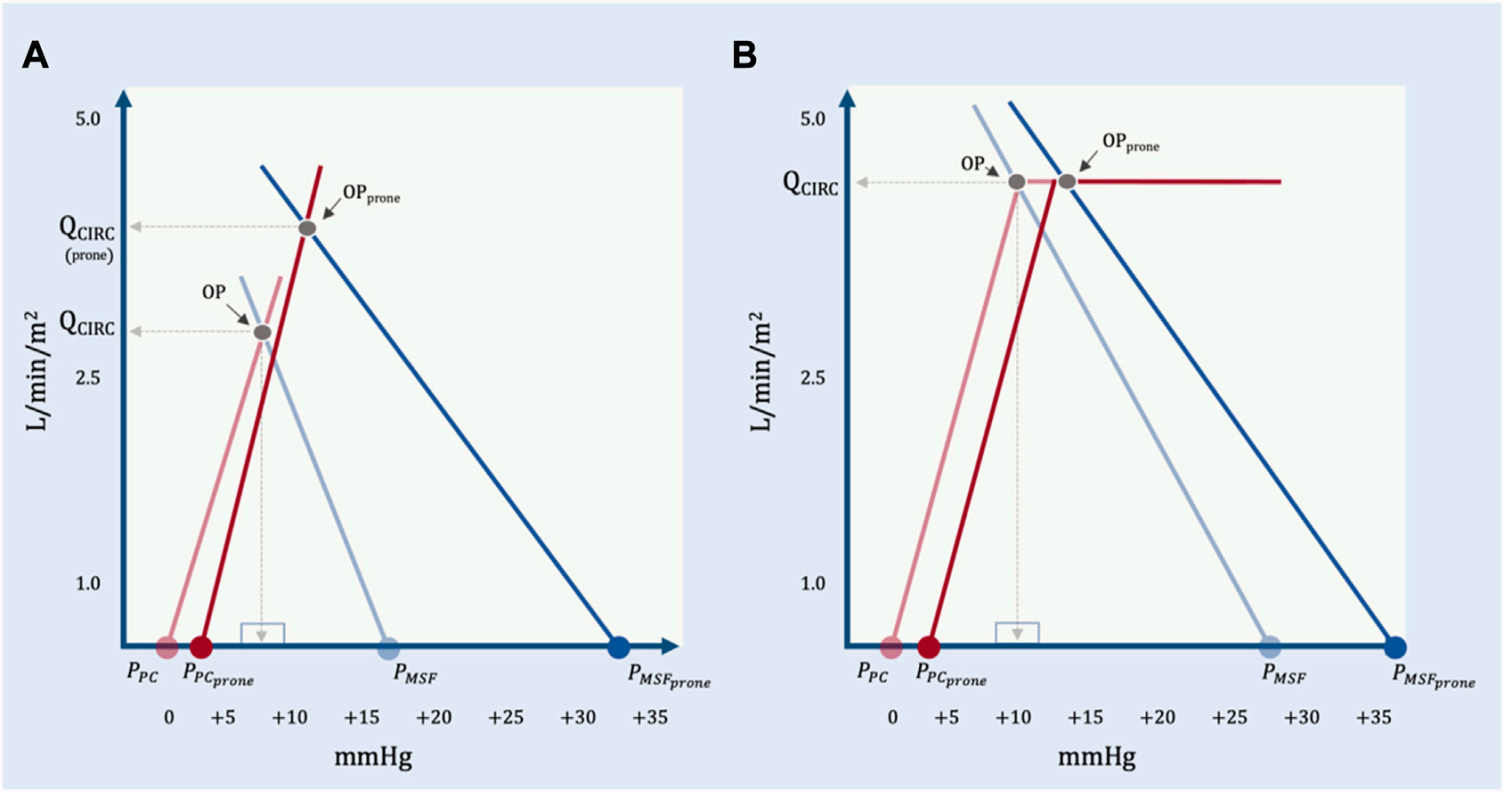
The geometrical model applied to representative data from Lai et al. **(A)** The effect of prone position on a preload responsive patient. At baseline, P_PC_ is estimated by assuming a mean airway pressure of 15 mmHg, an E_CW_/E_RS_ ratio of 0.2 and a pleural pressure (P_PL_) at functional residual capacity of—2.5 mmHg. With prone position, the P_MSF_ rises much more than P_PC_. There is an increase in R_VR_ and an assumed decrease in R_cardiac_ due to reduced pulmonary vascular resistance. The operating point with prone position (OP_prone_) leads to an increase in total blood flow (Q_CIRC_) and increased right atrial pressure (P_RA_). By this model, P_RA_ does not determine Q_CIRC_; both P_RA_ and Q_CIRC_ are determined by P_MSF_, P_PC_, R_cardiac_ and R_VR_. **(B)** Prone position in a preload unresponsive patient at baseline. P_PC_ in prone is estimated by assuming a mean airway pressure of 15 mmHg, and E_CW_/E_RS_ ratio of 0.5 and a P_PL_ at functional residual capacity of—2.5 mmHg. With cardiac limitation, only a significant change in R_cardiac_ would increase Q_CIRC_.

When patients are not ‘cardiac limited,’ the operating point may be either ‘unlimited’ or ‘venous limited.’ In the study of Jozwiak and colleagues, imaging of the great veins was not reported, but those patients who were preload responsive were unlikely to have great vein collapse (i.e., venous ‘waterfall’) given that their average, baseline P_RA_ was relatively high (i.e., 15 mmHg) with increased RVEDA/LVEDA ratios. Thus, based on equation 11 above, the change in Q_circulatory_ was probably subject to all of: P_MSF_, P_PC_, R_cardiac_ and R_VR_. Given what we know from Lai and colleagues, prone position likely increased P_MSF_; P_PC_ may have increased less than the rise in P_PL_ because of reduced pericardial restraint and R_cardiac_ fell due to diminished pulmonary vascular resistance. Each of these effects raise Q_circulatory_, presumably offsetting heightened R_VR_ with prone (Figure 10).

It is also possible for preload responsive patients to be ‘venous limited’ as described by equation 13 above. When the operating point lies on the flat portion of the venous return curve (i.e., below P_CRIT_) then R_cardiac_ ceases to affect Q_circulatory_. Said another way, blood flow is determined solely by peripheral venous factors. When ‘venous limited’, volume status is likely a crucial determinant of the hemodynamic response to prone position based on the model of Takata and Robotham described above (Takata et al., 1990). A zone 3 abdomen might have a stable or enhanced P_MSF_ relative to P_CRIT_ and blunt any increase in R_VR_ (i.e., stable or increased Q_circulatory_), while a zone 2 abdomen would diminish P_MSF_ relative to P_CRIT_ and favour elevated R_VR_ (i.e., stable or reduced Q_circulatory_). There is little data on ‘venous limited’ ARDS patients being placed in prone. In the study by Lai and colleagues, there were four ‘preload responsive’ patients who had no change (n = 3) or a decrease (n = 1) in Q_ciculatory_ when placed in prone position. These patients may have been venous limited, but this data was not collected. Given that at low trans-mural pressure, the great veins are very compliant (Bodson and Vieillard-Baron, 2012), generation of a hemodynamically-significant Starling resistor, i.e., ‘venous limitation,’ should lead to great vein collapse throughout most of the respiratory cycle. In a patient passive with the ventilator, collapse is an inspiratory event for the SVC and expiatory event for the IVC. Collecting this data with ultrasound before and after pronation could help delineate this hemodynamic phenotype.

Finally, it is possible to be both venous and cardiac limited simultaneously, in other words, the operating point is on both the flat portion of the venous return and cardiac function curves concurrently. This might happen in states of high P_CRIT_ (e.g., high PEEP, high subdiaphragmatic pressure) coupled with depressed cardiac function. In the setting of ARDS, this could be a syndrome of alveolar over-distension (Jardin and Vieillard-Baron, 2003). Prone position in such a patient might reduce Q_circulatory_, especially if the patient is hypovolemic. Managing this hemodynamic phenotype might involve PEEP titration to reduce P_CRIT_ and enhance cardiac function as this could move the operating point onto steep portions of the venous return and cardiac function curves.

## Conclusion

At equilibrium, the intersection of venous return and cardiac function generates the hemodynamic operating point. The operating point and both of its coordinates (i.e., P_RA_ and Q_circulatory_) are dependent variables. The independent variables of the system are the P_MSF_, resistance to venous return, cardiac function and the pressure surrounding the right atrium. These are not new principles; however, clinical physiology can be muddied in terms of how dependent and independent variables are discussed. A simplified geometrical model was presented to clarify the mechanisms of blood flow at equilibrium founded on Guyton’s model of the circulation; this focuses the clinician on how interventions in the ICU (e.g., prone position) might affect hemodynamics. Recent mechanistic investigations into the circulatory consequences of prone position have been reported. These findings were incorporated into the simplified geometrical model with emphasis on the link between cardiac and respiratory physiologies. The pericardial pressure is one nexus binding the heart and the lungs; so too are changes in cardiac function from pulmonary vascular recruitment. Measuring ‘preload responsiveness’ locates the system’s operating point; this helps predict the hemodynamic response to any intervention in the ICU, including the decision to prone a patient with ARDS.

## Data Availability

The original contributions presented in the study are included in the article/supplementary material, further inquiries can be directed to the corresponding author.
