# Addendum: A framework for heart-lung interaction and its application to prone position in the acute respiratory distress syndrome

**DOI:** 10.3389/fphys.2026.1901566

**Published:** 2026-07-29

**Authors:** Jon-Emile S. Kenny

**Affiliations:** 1 Health Sciences North Research Institute, Sudbury ON, Canada; 2 Flosonics Medical, Toronto, ON, Canada

**Keywords:** heart-lung interactions, hemodynamics, prone position, acute respiratory distress syndrome, venous return, fluid responsiveness

In 2023, “A Framework for Heart-Lung Interaction and its Application to Prone Position in the Acute Respiratory Distress Syndrome” was published in *Frontiers in Physiology* (Kenny 2023). This manuscript outlined a Geometrical Model (GM) of the circulation based upon Guyton’s principles. The GM solved for total blood flow (Q_CIRCULATORY_) as the dependent variable based upon the following independent variables: venous return conductance and resistance (i.e., G_VR_ and R_VR_, respectively), cardiac conductance and resistance (i.e., G_CARDIAC_ and R_CARDIAC_, respectively), mean systemic filling pressure (P_MSF_), and pericardial pressure (P_PC_).

Although Equation 8 was ultimately correct and the conclusion of the GM unchanged, the derivation was imprecise between Equations 6, 7 in the original manuscript, as per below:

(6) $$P_{MSF}-\frac{Q_{circulatory}}{{\text{G}}_{VR}}=\frac{Q_{circulatory}}{{\text{G}}_{cardiac}}+P_{PC}$$

(7) $$\frac{P_{MSF}}{{\text{Q}}_{circulatory}}-\frac{1}{{\text{G}}_{VR}}=\frac{Q_{circulatory}}{{\text{G}}_{cardiac}}+P_{PC}$$

(8) $$\frac{P_{MSF}-P_{PC}}{{\text{Q}}_{circulatory}}=\frac{1}{{\text{G}}_{cardiac}}+\frac{1}{{\text{G}}_{VR}}$$

From Equations 6, 7 above, only the left-hand side was divided by Q_CIRCULATORY_. Not shown between Equations 7, 8 above was that the right-hand side was also divided by Q_CIRCULATORY_ and then re-arranged *all in one step*.

However, this lacks mathematical detail and appears imprecise; a fuller derivation should have been as follows:

(6) $$P_{MSF}-\frac{Q_{circulatory}}{{\text{G}}_{VR}}=\frac{Q_{circulatory}}{{\text{G}}_{cardiac}}+P_{PC}$$

(7) $$\frac{P_{MSF}}{{\text{Q}}_{circulatory}}-\frac{1}{{\text{G}}_{VR}}=\frac{1}{{\text{G}}_{cardiac}}+\frac{P_{PC}}{{\text{Q}}_{circulatory}}$$

(8) $$\frac{P_{MSF}-P_{PC}}{{\text{Q}}_{circulatory}}=\frac{1}{{\text{G}}_{cardiac}}+\frac{1}{{\text{G}}_{VR}}$$

Though Equation 8 is unchanged, this ‘missing step’ should have been elaborated more clearly in the original manuscript. Drs. Jukka Takala and Per Werner Möller are thanked for bringing this to light and their many other contributions to the field of clinical hemodynamics.

